# DNA Regions Responsible for Maintenance of *Shingobium* Plasmid pYAN-2

**DOI:** 10.1264/jsme2.ME13135

**Published:** 2014-01-17

**Authors:** Hiroe Hayashi, Yasurou Kurusu

**Affiliations:** 1 United Graduate School of Agricultural Science, Tokyo University of Agriculture and Technology, 3–5–8 Saiwai-cho, Fuchu-shi, Tokyo 183–8509, Japan; 2 Department of Bioresource Science, Ibaraki University College of Agriculture, 3–21–1 Chuou, Amimachi, Ibaraki 300–0393, Japan

**Keywords:** sphingomonad, plasmid, partition, pSC101

## Abstract

We have identified and analyzed two DNA regions responsible for stable maintenance of a plasmid in the genus *Sphingomonas* and *Escherichia coli*. A 37 bp fragment, upstream of the *repA* gene, is required for stable maintenance of the low-copy-number small plasmid pYAN-2 (4,687 bp) from *Sphingobium yanoikuyae*. It does not encode any significant protein sequence and has one direct repeat for possible secondary structures. Moreover, a 70 bp fragment, upstream of the above sequence, completely stabilized the unstable pSC101 plasmid in *E. coli*.

Plasmids are, along with integrative and conjugative elements and phages, the major carrier platforms for horizontal gene transfer. Being non-essential extra-chromosomal pieces of DNA, plasmids need to evolve a number of strategies that allow them to persist in a growing bacterial population. The faithful inheritance of plasmids does not rely extensively on host-encoded factors. Rather, it is ensured by three different classes of plasmid-encoded maintenance functions: post-segregational killing systems (4, 7), multimer resolution systems (3), and partition systems as below.

Large low-copy-number bacterial plasmids encode partition systems that ensure active segregation of plasmid copies prior to cell division. Most partition systems consist of three elements: an NTPase (generally ATPase), a DNA-binding protein, and a centromere (9). DNA-binding protein, generically termed ParB, interacts with the centromere, *parS*, to create a partition complex. The NTPase, ParA, is recruited by the partition complexes to move sister plasmids toward opposite cell poles. Most partition systems are found in large or giant plasmids, but there have been no studies on the stabilization of small low-copy-number plasmids.

The genus *Sphingomonas* has received increasing attention because it includes various xenobiotic-degrading bacteria. Members of this genus are able to degrade compounds such as polycyclic aromatic hydrocarbons, chlorinated and sulfonated aromatics, herbicides, aromatic ethers, and polyethylene glycol (1). There have been several reports indicating that giant plasmids may be important in the degradation of xenobiotic compounds by *Sphingomonas* strains. In *Novosphingobium aromaticivorans* F199 and *Sphingomonas* sp. KA1, genes encoding the pathways for degrading biphenyl, naphthalene, *m*-xylene, and *p*-cresol have been detected on megaplasmids (15, 17). Moreover, strains of the genus *Sphingomonas* have a unique characteristic: they contain glycosphingolipids, which are ubiquitous in eukaryotic cell membranes (8). When *Sphingomonas* sp. A1 assimilates a macromolecule (alginate), a mouth-like pit (0.02–0.1 μm) is formed on the cell surface through reorganization and/or the fluidity of the pleats, causing extracellular alginate to be concentrated in the pit (6). This pit-dependent system of importing macromolecules was reported for the first time in a prokaryote, but appears to be the origin of endocytosis and phagocytosis in eukaryotes. Genetic manipulation of the genus *Sphingomonas* is necessary to improve the ability of these bacteria to degrade xenobiotic compounds and to determine the unique mechanisms involved in degradation, but no plasmid suitable for genetic manipulation of *Sphingomonas* has yet been developed. Some vector systems in sphingomonads with a broad-host-range plasmid or a cryptic giant plasmid have been reported by the conjugation method (1, 13). We previously reported the isolation and characterization of three small plasmids, pAMI-1 from *Sphingobium amiense* JCM 11777 (16) and pYAN-1 and pYAN-2 from *Sphingobium yanoikuyae* JCM 7371 (14), along with the development of a transformation system. In this report, we identified and characterized two non-coding DNA regions for a plasmid maintenance system from pYAN-2 in the genus *Sphingomonas* and in *Escherichia coli*.

First, we constructed a shuttle plasmid, pYAN2-A, between *E. coli* and *Sphingomonas*, with the regions surrounding the *repA* gene from pYAN-2 amplified by PCR using the following primers: 5′-AAGAATTCCTGATTG GGTGCTCTG-3′ (forward, *Eco*RI site underlined) and 5′-AAGGATCCTCCGCTAATCTAC-3′ (reverse, *Bam*HI site underlined) (the fragment from 2,855 bp to 4,367 bp in Fig. 1). The PCR fragment of pYAN-2 as a template was digested with *Eco*RI and *Bam*HI, and then inserted into the *Eco*RI/*Bam*HI-digested *E. coli* plasmid pHSG398, which harbors a chloramphenicol (Cm) resistance gene, and the result was designated pYAN2-A. As for a host strain, we chose *Novosphingobium capsulatum* JCM 7452 because no plasmid was detected in the cell (data not shown). This plasmid was transformed into *N. capsulatum* JCM 7452 by electroporation, as follows: Cells from 50-mL cultures of *Sphingomonas* strains (optical density at 660 nm = 0.7 to 0.8) were collected by centrifugation and washed twice with 10 mL of chilled 10% glycerol. The cells were resuspended in the same buffer to a final volume of 100 μL mixed with plasmid DNA (1 μg) and placed in 0.2 cm cuvettes. Electroporation was performed using a Gene Pulser (Bio-Rad, Hercules, CA) with a single pulse at 25 μF and 2.5 kV. The cells were allowed to grow in (write in full) (LB) for 2 h and then were spread on selection plates (15 μg mL^−1^ Cm).

Next we evaluated plasmid maintenance in the host population, as follows: Cells harboring plasmids were inoculated into LB supplemented with selective antibiotics and grown at 30°C to the stationary phase. At this point, cultures were diluted 10^5^-fold in fresh LB without antibiotics and grown for about 10 generations. Samples of each culture were taken at the beginning and the end of growth, diluted, spread on LB agar plates without antibiotics, and grown to 100–300 colonies per plate. The phenotypes of 100 colonies from each plate were examined by transferring them with toothpicks to selection plates containing antibiotics. Cell concentrations were determined before and after cultivation. Results are averages of three independent experiments. As a result, pYAN2-A was stably maintained in *Sphingomonas* cells for at least 40 generations in the absence of selection (Fig. 2). To determine the minimal region necessary for plasmid stabilization, we constructed various deletion plasmids by the same method as used for pYAN2-A (Fig. 1). All plasmids were constructed by PCR using the above reverse primer and various forward primers (5′-AAGAATTCGCGCCCTTCT TGTTCATAT-3′ for pYAN2-B containing the fragment from 3,081 bp to 4,367 bp, 5′-AAGAATTCGTTCATATA GTTCTT-3′ for pYAN2-C containing the fragment from 3,092 bp to 4,367 bp, and 5′-AAGAATTCGAAAAATG GCCTGC-3′ for pYAN2-D containing the fragment from 3,118 bp to 4,367 bp, *Eco*RI sites underlined). Two plasmids (pYAN2-B and pYAN2-C) successfully transformed *Sphingomonas* cells, but plasmid pYAN2-D could not (Fig. 1). These results suggested that the replication function of pYAN-2 is located within a 3,092 bp to 4,367 bp fragment. To determine the minimal region necessary for plasmid maintenance, we examined the stability of various deletion plasmids under nonselective culture conditions. pYAN2-B plasmid as well as pYAN2-A was stable but pYAN2-C was not (Fig. 2). These results indicated that the plasmid maintenance function was located within the DNA region from 3,081 bp to 3,118 bp of plasmid pYAN-2. The nucleotide sequence of the above DNA region is shown in Fig. 1. Genetyx software (Genetyx, Tokyo, Japan) was used to analyze the DNA sequence for possible open reading frames and showed that no complete protein was found in 37 bp fragment of pYAN-2. However, for possible secondary structures, one direct repeat was detected (Fig. 1). Several reports have shown that the partition function, termed *par*, of pSC101 in *E. coli* (12) or pLS11 (2), also referred to as pPOD2000 (5) in *Bacillus subtilis*, restores the partition ability only in *cis*. No encoded protein was found in either the 167 bp fragment from pLS11 (2) or the 375 bp fragment from pSC101 (10). There was no significant sequence or secondary structure homology between the 37 bp fragment of plasmid pYAN-2 and the 375 bp fragment of pSC101 or the 167 bp fragment of pLS11 at the nucleotide level.

Since even plasmids lacking the partition mechanism might be maintained relatively stably as multi-copy-number plasmids, we measured the difference in the copy number between plasmid pYAN2-B and plasmid pYAN2A-C as the amount of plasmid DNA relative to the amount of chromosomal DNA by our previous Southern blotting method (16). The copy number of the stable plasmid pYAN2-B was similar to that of the unstable plasmid pYAN2-C and each copy number was on average 1–2 (±1) per chromosome (data not shown). Moreover, the copy number of the stable plasmid pYAN2-A was also 1 or 2 plasmids per chromosome (data not shown), and pYAN-2, as well as pAMI-1, was stringently regulated for plasmid replication. Although the possibility of whether *repA* expression by the 37 bp fragment affects the copy number of pYAN-2 is still unknown, we concluded that the stability of pYAN-2 seemed to be due to a plasmid maintenance mechanism encoded by this fragment.

To test whether the *par* region of pYAN-2 could function in other bacteria, such as *E. coli*, we constructed an unstable *E. coli* plasmid using a low-copy-number plasmid. First, we constructed the unstable pMW119 (19) (based on plasmid pSC101) plasmid. The region surrounding the *par* gene from pMW119 was amplified by PCR using the following primers: 5′-AACCATGGGCTTGCGAGG-3′ (forward, *Nco*I site underlined) and 5′-AACCATGGTTCGGATTATC-3′ (reverse, *Nco*I site underlined) (the fragment from 341 bp to 57 bp in Fig. 3). The PCR fragment was digested with *Nco*I, re-ligated to eliminate the *par* gene, and the result was designated pMW119-Δ*par*. pMW119-Δ*par* was quite unstable in *E. coli* DH5α [(ϕ*80*d*lacZ*ΔM15) *endA*1 *recA*1 *hsdR*17(r^−^m^−^) *supE*44 *thi*-1 λ^−^*gyrA relA*1 F^−^ Δ(*lacZYA*-*argF*) U169] cells for at least 20 generations in the absence of selection (Fig. 3). To test whether the 37 bp region of pYAN-2 is able to stabilize plasmid pMW119-Δ*par*, we constructed plasmid pMWY2-A as follows: a DNA fragment including 37 bp region was amplified by PCR using the following primers: 5′-AACCATGGCTGGTGGCTCTGAGGC-3′ (forward, *Nco*I site underlined) and 5′-AACCATGGGAG CCGCGAGGG-3′ (reverse, *Nco*I site underlined) for pYAN-2 (the fragment from 2,958 bp to 3,117 bp in Fig. 3), and the PCR fragment DNA were digested with *Nco*I and then inserted into *Nco*I-digested pMW119-Δ*par* plasmid (Fig. 3). Plasmid pMWY2-A was slightly stabilized for 20 generations under non-selective culture conditions (Fig. 3). Next, we constructed the pMWY2-B containing upstream region of the above fragment as follows: a *par* region was amplified by PCR using the following primers: 5′-AACCATGGCTGAATGACGCTGAAGG-3′ (forward, *Nco*I site underlined) and 5′-AACCATGGGCCTGAACTATA AGAACTATATGAA-3′ (reverse, *Nco*I site underlined) for pYAN-2 (the fragment from 2,888 bp to 2,958 bp in Fig. 3), and the PCR fragment DNA were digested with *Nco*I, and then inserted into *Nco*I-digested pMW119-Δ*par* plasmid (Fig. 3). Plasmid pMWY2-B, as well as parent plasmid pMW119, was maintained in population for 20 generations under non-selective culture conditions (Fig. 3). Also, the copy-number levels between plasmids pMW119 and pMWY2-B in *E. coli* were similar (data not shown). To exclude the possibility that this fragment contains the resolution function of plasmid multimers (3), we checked DNA forms of three plasmids (pMW119, pMW119-Δ*par* and pMWY2-B) by electrophoresis from *E. coli recA*+ as a host and they all formed a plasmid-dimer (data not shown). These results suggested that a 70 bp fragment, upstream of the 37 bp region from pYAN-2, could function in *E. coli*. The nucleotide sequence of the above DNA region is shown in Fig. 3. We analyzed the DNA sequence for possible open reading frames and showed that no complete protein was found; however, one direct repeat and one inverted repeat were detected for possible secondary structures (Fig. 3). Moreover, there was no specific sequence similarity between the *par* locus of pSC101 (18) and the 70 bp region in pYAN-2 (Fig. 3). Meacock and Cohen proposed that the distribution of plasmid molecules (in the case of pSC101) between daughter cells at cell division is mediated by the interaction of this DNA locus with other cellular components of the partitioning system, such as the cytoplasmic membrane, and might be initiated by duplication of the *par* locus (10). Wahle and Kornberg showed that *par* of pSC101 is the major DNA gyrase-binding site and concluded that DNA gyrase, while involved in the partition function, may not affect plasmid stability through its supercoiling activity or by influencing DNA replication (18). On the other hand, Miller and Cohen showed that both DnaA and DnaB mediate pSC101 partitioning independently of their role in DNA synthesis (11). These mechanisms may also underlie the plasmid maintenance functions conferred by a 70 bp region of pYAN-2. We are also interested in plasmid maintenance, especially concerning the interaction of the DNA locus with the cytoplasmic membrane, and additional studies are now in progress.

In conclusion, we identified and characterized an efficient plasmid maintenance system for plasmids in the genus *Sphingomonas* and *E. coli*. Although it is unclear why pYAN-2 has two loci for the plasmid maintenance function, we believe that this will facilitate the molecular design of high-stable cloning vectors for the expression of foreign genes in sphingomonads for industrial purposes. Moreover, these data will contribute to analyzing the molecular evolution of environmental plasmids carrying various xenobiotic-degrading enzyme genes.

## Figures and Tables

**Fig. 1:**
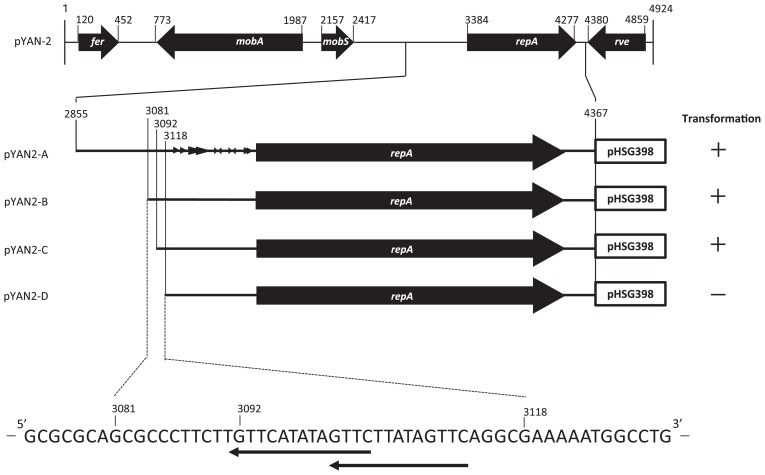
The ability to transform *Sphingomonas* with deletion derivatives of pYAN2-A. The nucleotide sequence data of pYAN-2 appear in the DDBJ, EMBL, and Genbank nucleotide sequence databases under accession no. AB265741. Functional descriptions of the five genes are as follows: *fer*, 2Fe-2S ferredoxin; *mobA*, putative MobA/MobL family protein; *mobS*, mobilization protein; *repA*, replication protein; *rve*, transposase. Plasmid construction and the transformation method are described in the text. Arrowheads:←, direct repeats; →←, inverted repeats.

**Fig. 2:**
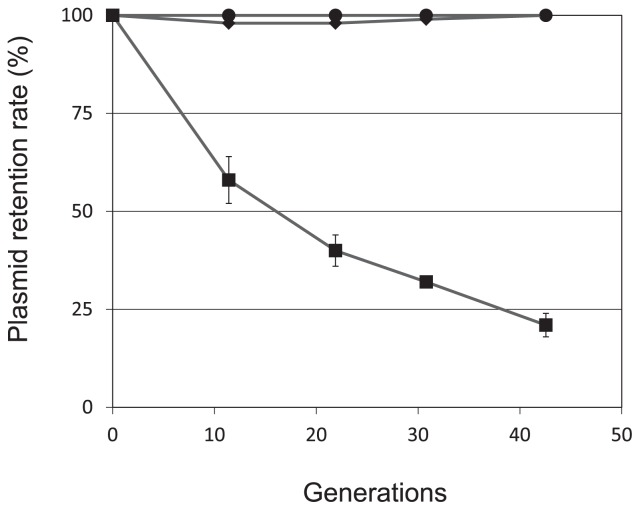
Stability of various plasmids under nonselective conditions. The stability test is described in Materials and Methods. Plasmid retention rate was obtained from 10, 20, 30, and 40 generations of growth. Symbols: ● pYAN2-A; ◆ pYAN2-B; ■ pYAN2-C.

**Fig. 3:**
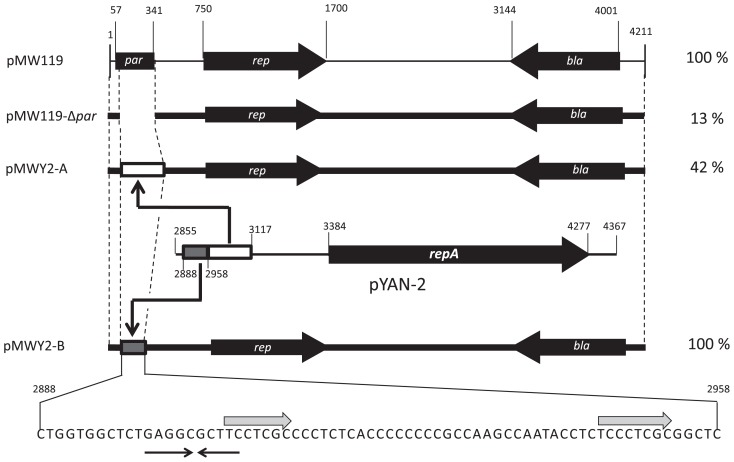
Construction and stability of pMW119Δ*par*, pMWY2-A, and pMWY2-B. Plasmid construction and the stability test are described in the text. Plasmid retention rate was obtained from about 20 generations of growth. Arrowheads: →←, inverted repeats; ⇒, direct repeats.
